# Improving the Detectability of Microplastics in River Waters by Single Particle Inductively Coupled Plasma Mass Spectrometry

**DOI:** 10.3390/nano13101582

**Published:** 2023-05-09

**Authors:** Celia Trujillo, Josefina Pérez-Arantegui, Ryszard Lobinski, Francisco Laborda

**Affiliations:** 1 Group of Analytical Spectroscopy and Sensors (GEAS), Institute of Environmental Sciences (IUCA), University of Zaragoza, Pedro Cerbuna 12, 50009 Zaragoza, Spain; ctrujillo@unizar.es (C.T.); jparante@unizar.es (J.P.-A.); 2 IPREM UMR 5254, CNRS, E2S UPPA, Université de Pau et des Pays de L’Adour, Hélioparc, 64053 Pau, France; ryszard.lobinski@univ-pau.fr; 3 Chair of Analytical Chemistry, Department of Chemistry, Warsaw University of Technology, ul. Noakowskiego 3, 00-664 Warszawa, Poland

**Keywords:** single particle detection, microplastics, river water, ICP-MS

## Abstract

Detection of microplastics in environmental samples requires fast, sensitive and selective analytical techniques, both in terms of the size of the microparticles and their concentration. Single particle inductively coupled plasma mass spectrometry (SP-ICP-MS) allows the detection of plastic particles down to ca. 1 µm and down to concentrations of 100 particles per mL. In SP-ICP-MS, detection of carbon-containing particles is hampered by the presence of other forms of carbon (carbonates, organic matter, microorganisms…). An acidic pre-treatment of river water samples with 10% (*v*/*v*) nitric acid for 24 h allowed the reduction of the presence of dissolved carbon to ultrapure water levels and the digestion of potential microorganisms in the samples, recovering polystyrene microparticles up to 80%. Carbon-containing particles were detected in most of the samples analysed from Spanish and French Pyrenean rivers. The presence of microplastics in these samples was confirmed by Raman microscopy and their morphology was defined by electron microscopy combined with energy-dispersive X-ray spectroscopy. The developed SP-ICP-MS method is suitable for the rapid screening of river waters for the presence of microplastics, which can then be analysed by inherently slower but more selective techniques (e.g., Raman microscopy).

## 1. Introduction

Plastic pollution is a global environmental threat leading to policies for the adequate use, waste management and recycling of plastics [1]. Plastics are classified in four groups according to their size: macroplastics, mesoplastics, microplastics and nanoplastics, although no standardised definitions are currently available [2]. Frias et al. [3] defined microplastics (MP) as “any synthetic solid particle or polymeric matrix, with regular or irregular shape and with size ranging from 1 μm to 5 mm”. In the case of nanoplastics, their size range tends to be fixed between 1–100 nm or below 1 μm. Gigault et al. [4] defined nanoplastics as “particles within a size ranging from 1 to 1000 nm resulting from the degradation of industrial plastic objects and can exhibit a colloidal behaviour”.

Nano- and microplastics circulate in the environment, resulting in the so-called “plastic cycle” [5]. The plastic cycle is currently made up of four “environmental compartments”: terrestrial, freshwater, seawater and atmospheric. These compartments are interconnected, with indistinct and permeable boundaries. The interactions between them are influenced by different weather and environmental conditions. Rivers accumulate a significant amount of plastics from urban wastewaters and play a prominent role in the “plastic cycle” as a transporter of MPs. Wastewater treatment plants have demonstrated their ability to remove about 99% of the plastics present, which results in 1% of the plastics being released back into the environment [6,7]. The concentration of plastics present in this 1% is strongly influenced by the influent plastic load or the type of WWTP [8,9]. It should also be noted that around 80% of the world’s wastewater is released untreated into rivers. It is therefore necessary to consider rivers as a dynamic reservoir of MPs [10].

The location of large cities and industrial areas close to rivers favours the presence of large amounts of MPs, stimulating studies in various rivers in Asia and Europe. Table 1 summarises these studies, including the techniques used for the analysis, the type of plastics detected, the concentration of MPs present and their size. As it can be seen, these studies covered the upper range of microplastics, being limited to sizes over 20 µm. Most studies relied on optical microscopy complemented with Fourier transform infrared (FT-IR) spectrometry for polymer identification, as well as FT-IR microscopy. Two studies considered the use of Raman microscopy or pyrolysis gas chromatography mass spectrometry (Pyr-GC-MS).

Whereas FT-IR microscopy is limited to particles over 10 µm, Raman microscopy can be used to detect plastic particles down to 1 µm [21]. Whereas these techniques provide information, both qualitative and quantitative, on a particle-by-particle basis, GC-MS techniques (pyrolysis and thermoextraction desorption) is used to quantify the average content of specific polymers in the samples. Recently, inductively coupled plasma mass spectroscopy (ICP-MS) has emerged as an alternative technique for the detection of carbon-containing particles when operated in single particle mode (SP-ICP-MS) [22,23]. The technique allows the detection of plastic particles down to ca. 1 µm with number concentrations of 100 particles per mL. The technique has been applied to the detection of microplastics in cosmetics [22], and to study their release from food packaging [22] as well as their aging by UV-light [24].

The determination of carbon by ICP-MS is hindered by low sensitivity and high background levels. The carbon sensitivity is limited by its low ionization efficiency (ca. 5%) because of its high ionization potential (11.26 eV), together with its low transmission in the ICP-MS interface due to space charge effects [25]. The background level is affected by the ubiquitous presence of carbon dioxide in air and water. As a result of these limitations, the attainable limits of detection are high, in the range of mg L^−1^ [26].

River water contains different forms of carbon, with around 60% present as inorganic carbon, while the rest corresponds to organic carbon, although this distribution can change depending on the river [27]. Inorganic carbon is mainly present as dissolved carbonates (HCO_3_^−^ and CO_3_^2−^), although the presence of particulate carbonates (CaCO_3_, MgCO_3_, etc.) or black carbon (soot) cannot be discarded. Organic carbon present in rivers includes different substances (proteins, carbohydrates, carboxylic acids, humic and fulvic acids, etc.) grouped together as dissolved organic matter (DOM), but particulate forms may be also present. The presence of these different forms of carbon in river water can pose a problem for the detection of microplastics and, consequently, they should be removed from samples prior to SP-ICP-MS analysis.

The aim of this work was to propose SP-ICP-MS as a screening technique for the detection of microplastics in river waters prior to their analysis by more selective techniques (e.g., Raman or FTIR microscopies, GC-MS). The specific features of river waters require a pre-treatment to eliminate other forms of carbon that can bias the results. The developed method was applied to the analysis of a number of samples from river basins in the north and south of the Pyrenees to obtain a first insight into the microplastic pollution of these areas.

## 2. Materials and Methods

### 2.1. Instrumentation

#### 2.1.1. Inductively Coupled Plasma Mass Spectrometry (ICP-MS)

A PerkinElmer NexION 2000B ICP-MS spectrometer (Toronto, ON, Canada) was used throughout. The sample introduction system consisted of an Asperon™ linear pass spray chamber (PerkinElmer, Toronto, ON, Canada), equipped with a flow-focusing nebulizer (Ingeniatrics, Sevilla, Spain) operated at argon nebuliser and make-up flow rates of 1.0 and 0.2 L min^−1^, respectively. A μDx Single Cell Autosampler (Elemental Scientific, Omaha, NE, USA) equipped with a syringe pump operating at 10 μL min^−1^ was used for sample introduction. ^13^C was monitored at a dwell time of 200 µs for a total acquisition time of 60 or 480 s. Argon of 99.999% purity was used. Data were processed with Syngistix NanoApplication version 2.5 and plotted with Origin 2019b.

#### 2.1.2. Raman Microscopy

A confocal Raman microscope WITec Alpha 300+ (Oxford Instruments, Abingdon, UK) was used. The objective used for sample visualization and spectral acquisition was ×100 (Oxford Instruments, Abingdon, UK). Raman scattering was excited with a 532 nm laser diode (Oxford Instruments, Abingdon, UK). The laser power used was 3.21 or 6.81 mW, depending on the sample. The spectra were obtained using 3 s of acquisition time and 25 collections for each spot of the samples analysed.

#### 2.1.3. Field Emission Scanning Electron Microscopy (FESEM)

A FESEM Carl Zeiss MERLIN™ (Nano Technology Systems, Jena, Germany) was used for visualization of samples. The microscope was equipped with an INCA 350 X-ray energy dispersive (EDX) system (Oxford Instruments, Abingdon, UK) for elemental analysis.

### 2.2. Standards

A suspension, obtained from BCR (Geel, Belgium), of reference latex spheres of 2 μm nominal diameter (RM165) made of polystyrene cross-linked with divinylbenzene and stabilised with a non-ionic surfactant was used. RM165 spheres have a certified diameter of 2.223 ± 0.013 μm and an approximate numerical concentration of 3.23 × 10^8^ L^−1^. A suspension of polystyrene microparticles (PS-MP) with a nominal diameter of 3 μm from Sigma (Saint Louis, MO, USA), with a certified diameter of 3.03 ± 0.09 μm, was also used. All dilutions were prepared in ultrapure water (Milli-Q Advantage, Molsheim, France) by accurately weighing (±0.1 mg) aliquots of the stock suspensions after 1 min sonication (Ultrasonic Cleaner Bath CE-5700 A, 42 KHz, 50 W). Particle suspensions were not stabilized by adding surfactants, in order not to increase their dissolved carbon content and hence the size detection limits.

Tartaric acid in 0.2% (*v*/*v*) HNO_3_ (Inorganic Ventures, Christiansburg, VA, USA) was used to prepare aqueous carbon solutions by dilution in 0.2% (*v*/*v*) HNO_3_. The suitability of this carbon standard for SP-ICP-MS analysis of microplastics was proven in a previous publication [22].

### 2.3. River Water Samples

River water samples were collected from rivers in France (Occitania and Nouvelle-Aquitaine) and in Spain (Aragon, Navarra, La Rioja, and Catalonia). The geographical locations of the sampling points are listed in Table S1 of the Supporting Information. Samples were taken in 500 mL glass bottles and stored at 4 °C.

### 2.4. Procedures

#### 2.4.1. SP-ICP-MS Analysis

The threshold criterion for discrimination of baseline readings from particle events was based on the following expression [28]:

(1) $$Y_{C}=Y_{B}+5\sigma_{B}$$

where $Y_{C}$ is the gross critical value expressed in counts, $Y_{B}$ is the mean baseline intensity and $\sigma_{B}$ its standard deviation, which was considered equal to $\sqrt{Y_{B}}$.

Critical values for the detection of particle events ($Y_{C}^{number}$) were calculated from the appropriate blanks (ultrapure water, 10% nitric acid, etc.) by using the following expression [29]:

(2) $$Y_{C}^{number}=Y_{N,B}^{}+2.33\sigma_{N,B}^{}$$

where $Y_{N,B}^{}$ is the mean number of particle events detected in the blank and $\sigma_{N,B}$ its standard deviation. All calculated critical values were rounded to the upper integer value.

Background equivalent concentrations (BEC) were calculated from the mean baseline intensity and the corresponding mass concentration calibration with dissolved carbon standards.

Size critical values ($X_{C}^{size}$) were calculated as equivalent diameters of spherical polystyrene particles expressed in nm [28]:

(3) $$X_{C}^{size}={\left(\frac{3\times 10^{8}\sigma_{B}}{\pi \rho F_{P}K_{ICPMS}K_{M}}\right)}^{1/3}$$

where ρ is the density of polystyrene (1.02 g cm^−3^); $F_{P}$ is the carbon mass fraction in polystyrene (0.9231); $K_{M}$ (${=AN}_{Av}/M_{M}$) is a factor related to the element measured, where A is the atomic abundance of the isotope considered (0.011 for ^13^C), $N_{Av}$ is the Avogadro number, and $M_{M}$ the atomic mass of the element (12.011 g mol^−1^); and $K_{ICPMS}$ is the detection efficiency, which represents the ratio of the number of ions detected versus the number of analyte atoms of the measured isotope introduced into the ICP. $K_{ICPMS}$ was estimated from a calibration with a dissolved standard of the element monitored, as described in Laborda et al. [22].

Particle diameters (d), expressed in nm, were calculated as:

(4) $$d={\left(\frac{6\times 10^{7}S_{P}}{{\pi \rho F_{P}K}_{ICPMS}K_{M}}\right)}^{1/3}$$

where $S_{P}$ is the net intensity of each particle event.

Particle number concentrations ($X^{number}$) were estimated from:

(5) $$S_{N}=\eta Q_{sam}t_{i}X^{number}$$

where $S_{N}$ is the net number of particle events detected in a sample ($Y_{N}^{}-Y_{N,B}^{}$), η the analyte transport efficiency, $Q_{sam}$ the sample flow rate and $t_{i}$ is the total acquisition time. The analyte transport efficiency (0.46) was determined by the frequency method [30] by using the RM165 polystyrene microparticle reference material. The sample flow rate (0.100 µL min^−1^) was measured gravimetrically.

#### 2.4.2. Acidic Pre-Treatment of River Water Samples for SP-ICP-MS Analysis

An aliquot of 300 μL of concentrated HNO_3_ was added to 2.7 mL of sample in a glass vial to reach an acid concentration of 10% (*v*/*v*). The mixture was stirred for 24 h at room temperature.

#### 2.4.3. Preparation of Ag-Labelled Bacterial Suspension

*E. coli* bacteria were suspended in 25 mL of Muller Hinton medium (Scharlau, Barcelona, Spain) together with 2% Tween 80 (Sigma, Saint Louis, MO, USA). The bacteria were exposed to 0.5 mg L^−1^ Ag(I) from AgNO_3_ (Sigma-Aldrich, St. Louis, MO, USA) for 24 h at 37 °C with agitation. After exposure, the supensions were centrifuged at 5300 rpm for 15 min (Digicen 20, Ortoalresa, Madrid, Spain). The supernatant was removed, and the pellet washed three times (5300 rpm 15 min) with phosphate buffer (Sigma-Aldrich, St. Louis, MO, USA). After washing, the bacteria were resuspended in 5 mL of phosphate buffer.

#### 2.4.4. FESEM Analysis

An aliquot of 2 mL of river water was filtered on alumina filters of 0.1 μm pore size (AnodiscTM 25, Cytiva, Amersham, UK) in a glass filtration system. Filters were dried at 60 °C for 15 min. After drying, filters were attached to the measurement support with carbon tape and coated with gold with a Leica EM SCD500 (Leica Microsystem, Vienna, Austria). The aim of the analysis was to obtain some insight into the shape of the carbon-containing particles present in the samples under study.

#### 2.4.5. Raman Microscopy Analysis

An aliquot of 2 mL of river water was subjected to the same filtration and drying procedure than for FESEM analysis. In this case, dried filters were deposited on a glass slide and stored until analysis.

The information about the composition of the analysed particles was obtained by comparing the measured spectra with reference spectra from KnowItAll^TM^ (Wiley, Hoboken, NJ, USA). The High-Quality Index (HQI) was used to evaluate the degree of agreement between the reference and the measured spectra. The aim of the analysis was to confirm the presence of microplastics in the samples under study.

## 3. Results

### 3.1. Preliminary Analysis of River Water Samples by SP-ICP-MS

Table 2 shows the results obtained for the direct analysis of the river waters with no dilution and an acquisition time of 60 s. The aim of this analysis was to obtain preliminary information about the presence of carbon-containing particles in the samples.

### 3.2. Acidic Pre-Treatment of River Water Samples

A pre-treatment based on the use of nitric acid was selected for removing dissolved and particulate carbonate species, as well as for oxidating natural organic matter and digesting microorganisms that could be present in the river water samples. Preliminary experiments were performed with 2 µm PS-MP suspensions by using the acid at concentrations ranging from 0.2 up to 50% (*v*/*v*) and heating the mixtures at 100 °C for a short period of time (5 min) or standing them at room temperature for 24 h. Particle recoveries ranged from 60 to 70% for nitric acid concentrations up to 10%, with significant loses over 70% when using 50% nitric acid and heating for 5 min (Table S2 in Supplementary Information).

**Figure 1:**
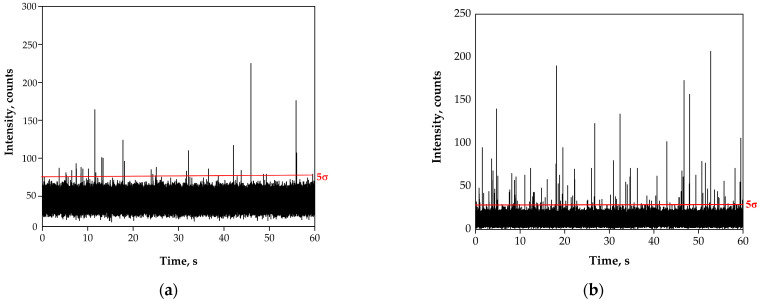

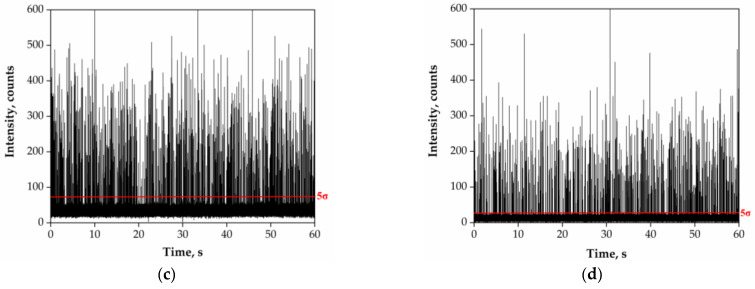
SP-ICP-MS time scans corresponding to (**a**) the original river water sample RW07, (**b**) the river water sample RW07 after pre-treatment with 10% (*v*/*v*) nitric acid for 24 h, (**c**) the river water sample RW07 spiked with 3 μm PS-MPs and (**d**) the river water sample RW07 spiked with 3 μm PS-MPs after pre-treatment with 10% (*v*/*v*) nitric acid for 24 h. Total acquisition time: 60 s. Red lines mark the 5σ threshold applied to each sample.

In view of the results obtained, it was decided to apply a pre-treatment based on the addition of nitric acid to the samples, to reach their final concentration of 10% (*v*/*v*), leaving them at room temperature for 24 h before analysis. Table 3 summarises the results obtained for the direct analysis of river water RW07 spiked with PS-MP of 2 and 3 µm. Parameters included in the table and their calculation are explained in detail in Section 3.2. Figure 1 shows the SP-ICP-MS time scans corresponding to the original river water RW07 and spiked with 3 μm PS-MPs, as well as after the pre-treatment with 10% (*v*/*v*) nitric acid for 24 h.

The efficiency of the acidic pre-treatment for the digestion of microorganisms present in the samples was tested by analysing bacterial suspensions by SP-ICP-MS (Figure S1). The carbon content per bacterium was proven to be too low for their quantitative detection by SP-ICP-MS, and silver-labelled suspensions of *E. coli*, obtained by culturing in the presence of AgNO_3_, were used. Internalization of Ag(I) was confirmed by subsequent analyses (unpublished results). The analysis of a bacterial suspension containing ca. 1 × 10^8^ CFU mL^−1^ showed 10,281 ± 101 events, which decreased to 93 ± 8 after the acidic pre-treatment (Figure S2).

**Figure 2:**
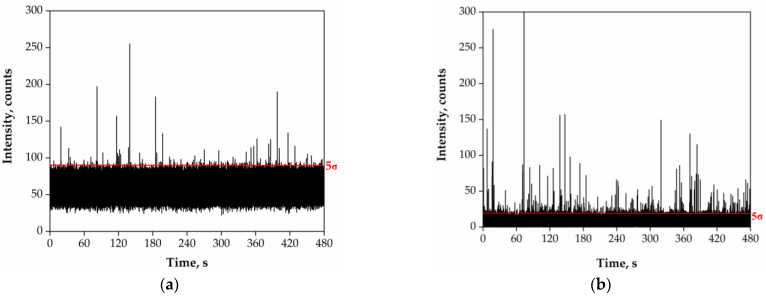
SP-ICP-MS time scans corresponding to (**a**) the original river water sample RW03, (**b**) after pre-treatment with 10% (*v*/*v*) nitric acid for 24 h. Total acquisition time: 480 s. The red lines mark the 5σ threshold applied to each sample.

In a next step, the samples with the highest number of events recorded (RW01, RW02, RW03, RW05, RW14, RW23, RW24, RW25 and RW27) were selected for a more in-depth analysis. The selected samples were analysed by SP-ICP-MS without dilution at a total acquisition time of 480 s, the maximum allowable time due to limitations of data storage of the ICP mass spectrometer used. The samples were also analysed after applying the acidic pre-treatment selected in Section 3.1. The results are shown in Table 4. Figure 2 shows time scans corresponding to sample RW03 without and with acidic pre-treatment.

### 3.3. Analysis of River Water Samples by Scanning Electron and Raman Microscopies

**Figure 3:**
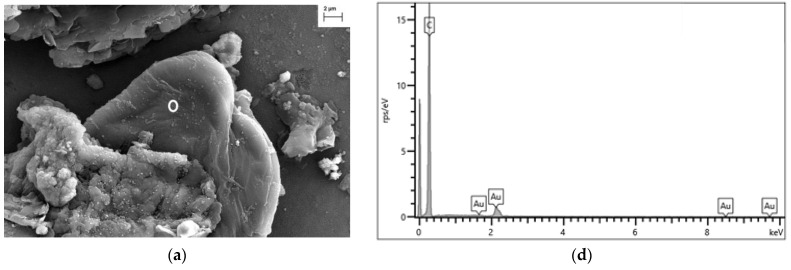

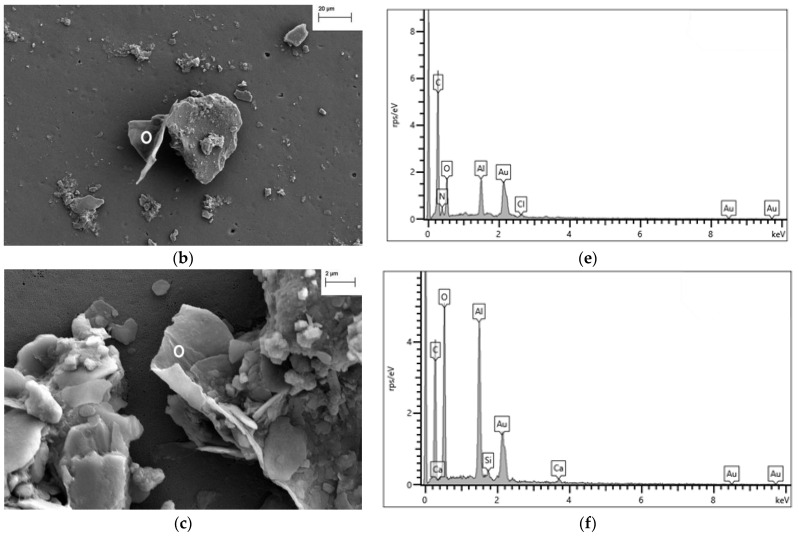
(**a**–**c**) FESEM images and (**d**–**f**) EDX spectra corresponding to particles visualised in river water samples. River water samples: (**a**,**d**) RW03, (**b**,**e**) RW02 and (**c**,**f**) RW25. White circles in FESEM images indicate the area analysed by EDX.

Three river water samples with the highest number of events detected by SP-ICP-MS (RW02, RW03 and RW25) were chosen to visualize particles according to their composition by FESEM-EDX. Sample preparation was carried out as described in Section 2.4.2. Figure 3 shows some of the particles visualised by FESEM in the river water samples, which could be considered as potential plastic particles due to the presence of carbon and/or oxygen in their EDX spectra.

**Figure 4:**
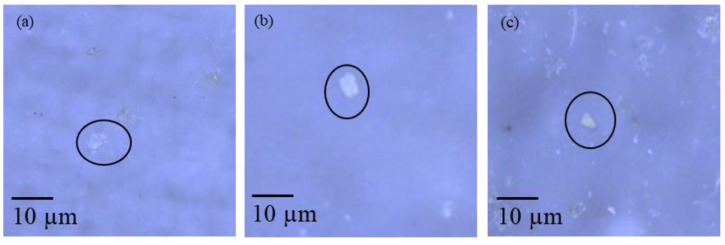
Optical microscopy images of particles from river waters samples RW01 (**a**), RW03 (**b**) and RW25 (**c**) analysed by Raman microscopy.

River water samples containing higher number concentrations of particles detected by SP-ICP-MS (RW01, RW02, RW03, RW23 and RW25) were selected for Raman microscopy analysis. Sample preparation was carried out as described in Section 2.4.3. About 20 particles from each filter were selected by visual inspection and the individual Raman spectra were recorded (Figure S3). Figure 4 shows images of some of the particles in the river water samples analysed by Raman microscopy and Table 5 summarises the results obtained.

## 4. Discussion

### 4.1. Acidic Pre-Treatment of River Water Samples

As stated in the Introduction, river waters contain different inorganic and organic carbon species, both in dissolved and particulate forms. Dissolved carbonates present in river waters originate from the reactions of the dissolved carbon dioxide on calcareous minerals from the river basin. Black carbon particles are due to incomplete combustion of fossil fuels and biomass; they originate from aerial deposition and are transported by run-off waters from the riverbanks [31,32]. The origin of dissolved and particulate organic matter is the degradation of vegetation and other organisms. Microorganisms (bacteria, microalgae, etc.) are naturally present in rivers and have to be considered as another form of carbon-containing particles [33,34].

The detection of microplastics by SP-ICP-MS can be affected by the presence of the carbon forms cited above. Firstly, the presence of DOM and dissolved carbonates will have an effect on the increase in the baseline in the recorded time scans, reducing the detectability of smaller microplastics. Secondly, microorganisms, insoluble carbonates and black carbon may be misidentified as microplastics because of their carbon content. Therefore, there is a need for sample pre-treatment to remove these forms of carbon from river water samples.

The rivers studied flow through basins which are largely made up of marl, gypsum, limestone, shale and siltstone [35]. Marl and limestone are rocks rich in calcium, magnesium and other carbonates. The waters under study contain relevant concentrations of inorganic dissolved carbon (100–200 mg HCO_3_^−^ L^−1^ [36,37]) and the presence of particulate carbonates should not be discarded. Note that the mass fraction of carbon in alkaline-earth carbonates is low (0.3–0.4), which means that only particles larger than ca. 20 μm could theoretically be detected by SP-ICP-MS. However, the nebulization efficiency of such particles is very low, which makes their detection by ICP-MS unlikely [22].

As the main sources of dissolved carbon in the samples are carbonate species, a pre-treatment based on the use of nitric acid was selected. Upon acidification, carbonate species were transformed into carbon dioxide and partially released from the solution. Alkaline earth carbonates would also dissolve if present. Results presented in Table 3 and Table 4 show that the application of a pre-treatment with 10% nitric acid for 24 h reduced the carbon content to blank levels, at BECs of ca. 25 mg L^−1^, while ensuring recoveries up to 83% for polystyrene microparticles. On the other hand, the size of the polystyrene microparticles did not show relevant variations when subjected to the acidic pre-treatment (Table 3 and Table S2), although the potential effect of the pre-treatment on other types of polymers should be considered in more in-depth studies.

Due to the oxidant properties of nitric acid, the oxidation of natural organic matter to carbon dioxide could also be promoted, as well as the digestion of microorganisms. The dissolved organic carbon in the rivers studied is between ca. 3–8 mg L^−1^ [36,37] and hence, its contribution to the total carbon content is low in comparison with the inorganic fraction. However, the occurrence of microorganisms was confirmed by FESEM, since bacteria were observed in some samples. The efficiency of the acidic pre-treatment for digestion of bacteria was checked by analysing suspensions of *E. coli* labelled with silver by SP-ICP-MS, confirming the capability of the pre-treatment to eliminate >99% of bacteria. Although the individual bacteria were not detected due to their small size (in the micrometer range) and their low carbon content, this would be not the case for bacterial agglomerates or microalgae, which have been successfully detected by SP-ICP-MS [38].

### 4.2. Analysis of River Water Samples by SP-ICP-MS

Preliminary direct analyses of the river waters showed higher baseline intensities in comparison with ultrapure water, which led to an increase in the size critical values, affecting the number of particles detected, as well as their size range. Under the measurement conditions, the best size critical value was 1.25 μm and 3 ± 2 particle events were detected in ultrapure water (Table 3). The criterion considered for the detection of particles according to their size was based on 5 times the standard deviation of the baseline (Equation (1)) [28,39]. It was applied indistinctly to the calculation of the critical value, used for the processing of the SP-ICP-MS data, and of the size detection limit, used as a figure of merit [28,39]. However, the detection of particles over the size critical value requires a different criterion based on counting statistics (Equation (2)) [29]. When no particles are detected in a blank, the critical value ($Y_{C}^{number}$) is 0; however, if particles are detected in the blank, Equation (2) must be used. As up to 3 particle events were detected in the blanks, a critical value of 8 events was applied to the samples listed in Table 2 and Table 4. Whereas the application of the number concentration critical value allows the confirmation of the presence of particles over the size critical value, the reporting of the number concentration results requires recording particle events over a limit of quantification. Under ideal counting conditions (when no particles are detected in a blank) the limit of quantification requires counting 100 particles [29]. In such a case, the number concentration limit of quantification is defined by [39]:

(6) $${LOQ}_{number}=\frac{100}{\eta Q_{sam}t_{i}}$$

More complex expressions were proposed for non-ideal blank number of events [29], but they will not be considered here for the sake of simplicity.

Samples analysed directly without pre-treatment (Table 4) showed baseline intensities ranging from 18 up to 57 counts, corresponding to BECs from 60 to 190 mg L^−1^. In view of the results summarised in Table 3, the application of a critical value of 8 events implies that all samples except RW16 would contain carbon bearing particles, although the concentrations shown could not be considered reliable, as they were below the ${LOQ}_{number}$, which was 1.6 × 10^7^ L^−1^.

In order to improve the detection capability of particles, the total acquisition time was increased from 60 to 480 s, reducing the ${LOQ}_{number}$ by a factor of 8. Under such conditions, size critical values from 1.26 for ultrapure water up to 1.84 μm in sample RW03 were obtained. Whereas this variation in the size critical value can be considered not significant from the point of view of size, it is relevant from the point of view of the number of particles detected. As can be seen in Table 4, even by increasing the total acquisition time, more than 100 particle events were recorded only in three samples (RW14, RW23 and RW24). When the acidic pre-treatment was applied, the baselines were reduced below 10 counts for all the samples, reaching, in some cases, the baseline of the procedure blank (RW01, RW02, RW23, RW24 and RW27). Under such conditions, the best available size critical values were achieved (ca. 1.25 μm) and hence the best overall detection limits. Figure 2 shows the decrease in the baseline following sample pre-treatment. The positive effect of reducing the baseline intensity is clearly seen in samples RW03 and RW05, in which the number of particles detected increased 5 times after pre-treatment. In other cases (RW14, RW23, RW24, RW25, RW27), the number of particle events detected remained similar, within a range of ±25%, whereas samples RW01 and RW02 showed a 50% reduction in the number of events previously detected in the untreated samples. These lower recoveries may be due to the characteristics of the river waters or the features of the particles detected, which deserve further studies. Note that the recovery studies performed with the PS microparticle standards should be considered as an approximation, because the nature and stability of the particles in river water are not the same as they are in the standards, which are stabilized by surfactants present in the original suspensions.

In any case, the results shown in Table 4 confirm the presence of carbon-containing particles in all the samples. From a quantitative point of view, the number concentrations reported for samples RW03, RW05, RW14, RW23, RW24 and RW25, analysed after acid pre-treatment, as well as those in samples RW14, RW23 and RW24, analysed directly, could be considered valid, as they were calculated from the detection of more than 100 events, the minimum limit of quantification in the counting process. However, considering that just microparticles in a narrow size range were detected by SP-ICP-MS, these number concentrations should be considered as semiquantitative minimum values. For these reasons, more in-depth correlation between the river waters analysed and the results obtained could not be carried out.

### 4.3. Analysis of River Water Samples by Electron and Raman Microscopy

The analysis by SP-ICP-MS of the river waters confirmed the presence of carbon-containing particles in the samples. Since SP-ICP-MS is used as a screening technique, the identification of these carbon-containing particles as microplastics requires the use of more selective techniques, capable of providing information about the specific chemical composition, on a particle-by-particle basis. FESEM in combination with EDX was selected to obtain information about the morphology of carbon-containing particles, whereas Raman microscopy was used to confirm the chemical identity of microplastics.

#### 4.3.1. FESEM-EDX Analysis

Identification of a particle as a potential plastic using FESEM requires obtaining additional information through the elemental spectra obtained by EDX. Plastics containing a hydrocarbon backbone (e.g., PE, PP, PS) will show a high intensity carbon peak (at 277 eV, Kα_1,2_ line), whereas those with functional groups containing oxygen (e.g., PET, Nylon) or any other heteroatom (e.g., PVC, PVF, PAN) will show additional peaks corresponding to those elements [40]. In any case, the use of EDX for plastic identification cannot be considered conclusive, since a soot particle would produce a spectrum with a peak at 277 eV.

All the spectra obtained for the particles in Figure 3 showed a significant carbon peak. In the case of Figure 3a, no other element was recorded. In Figure 3b,c, the presence of carbon and oxygen was confirmed, as well as that of calcium in Figure 3c, which might indicate calcium carbonate. The particles selected in Figure 3 represent potential plastic particles, which showed in all cases irregular shapes, which would correspond to microparticles obtained by the degradation of macroplastics [21].

In order to verify whether particles such as those visualised by FESEM correspond to plastic particles or not, it is necessary to apply a technique providing information about the chemical structure, such Raman microscopy.

#### 4.3.2. Raman Microscopy

The polymer assignments to the analysed particles were based on the quality parameter provided by the software. As a general rule, HQIs above 60% were considered, confirming the presence of plastic particles in the river waters, which was the main objective of the analysis.

The number of plastic particles detected in river water samples varied depending on the sample. Around 15% of the particles analysed were plastics corresponding to polylactic acid, PMMA, HDPE, PE, PVA and PP. The remaining 85% consisted of wool fibre, cellulose and various minerals (quartz, silica, pyrophyllite, muscovite, zeolite and malaquite, among others). The presence of these plastic particles in the waters is compatible with their uses. PVA is used as a polymeric adhesive, whereas PMMA, HDPE, PP and PE are used in the fabrication of a variety of consumer products. Polylactic acid is also used in many consumer goods industries (e.g., food packaging or hygiene products) [41,42] and in agriculture [42,43]. The use of this type of biopolymers is increasing, due to the European directive 2019/904,19 which prohibits the introduction of single-use plastic products on the market.

## 5. Conclusions

SP-ICP-MS has been applied for the first time to the detection of plastic particles in river waters. In comparison with other techniques applied in this field, SP-ICP-MS allows the detection of smaller particles than optical or FT-IR microscopy. Although its size detection capability is similar to that of Raman microscopy, the analysis times are shorter. Considering GC-MS techniques, SP-ICP-MS provides information on a particle-by-particle basis instead of a mean mass content of polymer.

SP-ICP-MS is known to suffer from some limitations for the analysis of microplastics. The size of microplastics that can be detected is limited to the range of a few micrometres, while their nebulisation depends on their size above 3–5 µm. However, as microplastics in the environmental waters occur as a size continuum, from nm to µm and above, SP-ICP-MS is a technique suitable for their screening. Nevertheless, the carbon-specific response and the lack of selectivity towards the different polymers requires its implementation in combination with Raman microscopy and/or GC-MS, becoming a valuable component of an analytical platform for the monitoring of plastic pollution in the environment. An acidic pre-treatment improves the detectability of plastic microparticles in river water samples due to the reduction of the dissolved carbon content. The presence of other carbon-containing particles, such as carbonates or micro-organisms, would not be detected by SP-ICP-MS due to their low carbon content, and would require much larger particles than those efficiently nebulised in ICP-MS. In any case, the nitric acid pre-treatment would dissolve carbonate microparticles and degrade microorganisms. Only black carbon microparticles, originating from atmospheric emissions due incomplete combustion of fuels and biomass, would be detected by SP-ICP-MS.

The presence of plastic microparticles was detected in most of the river waters analysed and the SP-ICP-MS results were complemented using Raman microscopy and FESEM-EDX. The study indicates microplastic pollution in the rivers on both the northern and southern sides of the Pyrenees.

## Figures and Tables

**Table 1:** Studies related to the occurrence of microplastics in rivers.

River	Country	Technique	Type of Plastic	Size	Plastic Concentration	Ref.
Citarum	Indonesia	Optical microscopy FTIR Raman microscopy	PET PS Cellophane Nylon PP PE	˂300 µm - >1000 µm	3.35 ± 0.54 m^−3^	[11]
Ticino	Italy	Optical microscopy FTIR microscopy	LDPE PET PP	>20 µm	33 ± 21 m^−3^	[12]
Biala Czarna Hancza	Poland	Optical microscopy	-	>0.04–4 µm	10.83 ± 3.96 L^−1^ 10.29 ± 3.90 L^−1^	[13]
Yangtze	China	Optical microscopy Raman microscopy	PP PE PA PS PVC PET PC	>0.11 µm	1.27 ± 0.83 L^−1^	[14]
Thames	United Kingdom	Optical microscopy FTIR	Rubber PVC PE	>0.5 mm - 5 mm	51 ± 10 L^−1^	[15]
Northern Dvina	Russia	FTIR	PE PP EEA	>0.5 mm	0.6–1.4 × 10^4^ km^−2^	[16]
Seine	France	Optical microscopy FTIR microscopy	PP PE PES	32–2528 µm	15.5 ± 4.9 L^−1^	[17]
Lis	Portugal	Optical microscopy FTIR FTIR microscopy	PP PVC PC Nylon	14–4726 µm	234 ± 398 m^−3^	[18]
Elbe Mulde	Germany	Optical microscopy Pyr-GC-MS	PE PP PS	>50 µm	15 ± 2 m^−3^ 0.33–1.19 mg m^−3^	[19]
Garone	France	Optical microscopy ATR-FTIR	PE PP PS	700 µm - 5 mm	0.15 ± 0.46 m^−3^	[20]

**Table 2:** Preliminary results of the analysis of river waters by SP-ICP-MS. Total acquisition time: 60 s. Mean ± standard deviation (n = 3). X_C_^size^: size critical value.

Sample	X_C_^size^ (μm)	Number of Particle Events Detected	Particle Concentration (×10^4^ L^−1^)
UP water	1.25	3 ± 2	-
RW01	1.44	38 ± 10	295 ± 32
RW02	1.44	40 ± 12	310 ± 25
RW03	1.53	28 ± 8	217 ± 21
RW04	1.48	10 ± 3	109 ± 12
RW05	1.49	28 ± 7	217 ± 15
RW06	1.55	22 ± 5	171 ± 20
RW07	1.63	17 ± 6	132 ± 19
RW08	1.54	15 ± 4	116 ± 26
RW09	1.41	16 ± 4	124 ± 15
RW10	1.47	19 ± 8	147 ± 10
RW11	1.39	13 ± 3	101 ± 9
RW12	1.45	17 ± 4	132 ± 9
RW13	1.51	10 ± 5	78 ± 7
RW14	1.47	27 ± 6	209 ± 19
RW15	1.46	24 ± 4	186 ± 13
RW16	1.52	6 ± 2	47 ± 5
RW17	1.46	20 ± 4	155 ± 20
RW18	1.48	19 ± 3	147 ± 27
RW19	1.47	16 ± 2	124 ± 30
RW20	1.49	12 ± 2	93 ± 20
RW21	1.35	20 ± 3	155 ± 32
RW22	1.31	18 ± 4	140 ± 16
RW23	1.37	42 ± 11	326 ± 42
RW24	1.37	27 ± 10	209 ± 21
RW25	1.31	43 ± 9	334 ± 44
RW26	1.50	11 ± 3	85 ± 6
RW27	1.35	29 ± 6	225 ± 30
RW28	1.32	16 ± 5	124 ± 10

**Table 3:** Mean diameters and particle recovery of river water samples subjected to pre-treatment with 10% (*v*/*v*) nitric acid. Plastic microparticles: 2 and 3 μm. River water sample: RW07. Mean ± standard deviation (n = 3 replicated measurements). Recovery tests were carried out in duplicate.

Sample	HNO_3_ (% *v*/*v*)	Mean Diameter (μm)	Particle Concentration (×10^6^ L^−1^)	Particle Recovery (%)
2 µm	-	2.12 ± 0.02	313 ± 10	-
RW07 + 2 µm	-	2.54 ± 0.03	227 ± 11	73
RW07 + 2 µm	10	2.23 ± 0.01	261 ± 5	83
3 µm	-	3.41 ± 0.03	220 ± 21	-
RW07 + 3 µm	-	3.41 ± 0.02	169 ± 19	77
RW07 + 3 µm	10	3.15 ± 0.03	130 ± 2	60

**Table 4:** Results from selected river waters analysed by SP-ICP-MS without and with acidic pre-treatment (10% HNO_3_, 24 h). Total acquisition time: 480 s. Mean ± standard deviation (n = 3 replicated measurements). BEC: Background equivalent concentration of carbon. X_C_^size^: size critical value.

Sample	Baseline Intensity (Counts)	BEC (mg L^−1^)	X_C_^size^ (μm)	Number of Particle Events Detected	Particle Concentration (×10^4^ L^−1^)
direct analysis					
UP water	6 ± 2	27	1.26	3 ± 2	-
RW01	45 ± 1	150	1.72	88 ± 10	188 ± 21
RW02	41 ± 2	137	1.70	84 ± 11	180 ± 24
RW03	57 ± 2	190	1.84	45 ± 15	96 ± 31
RW05	50 ± 3	167	1.82	41 ± 16	88 ± 34
RW14	46 ± 2	153	1.75	230 ± 21	490 ± 45
RW23	30 ± 1	100	1.62	110 ± 4	249 ± 9
RW24	18 ± 1	60	1.48	119 ± 10	253 ± 21
RW25	27 ± 1	90	1.58	95 ± 8	182 ± 17
RW27	20 ± 2	67	1.50	91 ± 18	194 ± 39
acidic pre-treament (10% HNO_3_ 24 h)					
Proc. blank	7 ± 1	23	1.25	2 ± 2	-
RW01	7 ± 1	23	1.28	50 ± 8	106 ± 18
RW02	7 ± 1	23	1.26	46 ± 5	97 ± 11
RW03	9 ± 1	30	1.33	256 ± 37	554 ± 78
RW05	9 ± 1	30	1.31	214 ± 49	470 ± 70
RW14	8 ± 1	26	1.32	164 ± 40	349 ± 84
RW23	7 ± 2	23	1.25	107 ± 11	227 ± 15
RW24	7 ± 1	23	1.27	104 ± 11	221 ± 23
RW25	6 ± 1	20	1.24	120 ± 19	277 ± 37
RW27	7 ± 1	23	1.27	93 ± 10	197 ± 22

**Table 5:** Identification of individual plastic particles detected by Raman microscopy after spectra processing with KnowItAll^TM^.

	KnowitAll^TM^	
Sample	Composition	HQI
RW01	Polylactic acid	76.2
	PMMA	73.9
	Polylactic acid	64.7
	Polylactic acid	65.3
	PMMA	65.1
RW02	HDPE	90.6
	Polylactic acid	66.4
	HDPE	91.4
RW03	PVA	73.1
	Polylactic acid	78.4
	PE	90.9
RW23	PVA	66.8
	PE	85.2
	PP	89.5
	PP	87.7
RW25	PMMA	87.9

## Data Availability

Data are contained within the article or Supplementary Material.

## Supplementary Materials

The following supporting information can be downloaded at: https://www.mdpi.com/article/10.3390/nano13101582/s1, Table S1: List of rivers studied and locations of the sampling points; Table S2: Recovery information of microparticles subjected to pre-treatments; Figure S1: Time scan corresponding to the analysis of bacteria; Figure S2: FESEM image of bacteria form river water filtered on glass fibre; Figure S3: Raman spectra of the plastic particles.

## Abbreviations

BEC: background equivalent concentration, EDX: X-ray energy dispersive spectroscopy, FESEM: field emission scanning electron microscopy, FT-IR: Fourier transform infrared spectrometry, GC-MS: gas chromatography-mass spectrometry, HDPE: high density polyethylene, LDPE: low density polyethylene, MP: microparticle, PA: polyamide, PAN: polyacrylonitrile, PC: polycarbonate, PE: polyethylene, PES: polyethersulfone, PET: polyethylene terephthalate, PMMA: polymethylmethacrylate, PMP: polymethylpentene, PS: polystyrene, PP: polypropylene, PVA: polyvinyl acetate, PVC: polyvinyl chloride, PVF: polyvinil fluoride, SP-ICP-MS: single particle inductively coupled plasma mass spectrometry.
