# Supplementary material for: Improving the Detectability of Microplastics in River Waters by Single Particle Inductively Coupled Plasma Mass Spectrometry
Source: Nanomaterials (Basel). 2023 May 9;13(10):1582. doi: 10.3390/nano13101582 (PMC10223556; doi:10.3390/nano13101582)
Supplement: Supplementary file 1 [file nanomaterials-13-01582-s001.zip › nanomaterials-2346649-supplementary.pdf]

Supplementary information

Improving the detectability of microplastics in river waters by single particle inductively coupled plasma mass spectrometry

Celia Trujillo [1], Josefina Pérez-Arantegui [1], Ryszard Lobinski [2,3] and Francisco Laborda [1] *

[1] Group of Analytical Spectroscopy and Sensors (GEAS), Institute of Environmental Sciences (IUCA), University of Zaragoza, Pedro Cerbuna 12, 50009, Zaragoza, Spain; ctrujillo@unizar.es
[2] Université de Pau et des Pays de L'Adour, E2S UPPA, CNRS, IPREM UMR 5254, Hélioparc, 64053, Pau, France; ryszard.lobinski@univ-pau.fr
[3] Chair of Analytical Chemistry, Department of Chemistry, Warsaw University of Technology, ul. Noakowskiego 3, 00-664 Warszawa, Poland; ryszard.lobinski@pw.edu.pl
* Correspondence: flaborda@unizar.es; Tel.: +34 976762252

2.3. River water samples.

Table S1. List of rivers studied and locations of the sampling points.

Sample identification	River	Location	Longitude	Latitude	Additional information
RW01	Segre	Torres de Segre	0º 30' 46,035'' E	41º32' 8,665'' N	rural area
RW02	Noguera Ribagorzana	Corbins	0º 41' 44,807'' E	41º 41' 42,562'' N	rural area
RW03	Clamor Amarga	Zaidín	0º 9′ 23,535″ E	41º 31' 32,533'' N	rural area
RW04	Cinca	Fraga	0º 8' 55,230'' W	41º 47' 29,677'' N	rural area
RW05	Alcanadre	Sariñena	0º 8' 55,230'' W	41º 47' 29,677'' N	rural area
RW06	Flumen	Albalatillo	0º 9' 42,000'' W	41º 44' 5,000'' N	rural area
RW07	Gállego	San Mateo de Gállego	0º 48′ 50,780'' W	41º 43′ 59,406'' N	rural area
RW08	Arba de Ríquel	Ejea de los Caballeros	1º 13' 18,312'' W	42º 7' 57,812'' N	rural area
RW09	Aragon Subordan	Javierregay	0º 42′ 19,17'' W	42º 37′ 47,026'' N	rural area
RW10	Aragón	Caparroso	1º 38' 31,757'' W	42º 21' 2,191'' N	rural area
RW11	Irantzu	Estella	2º 0' 6,691'' W	42º 38' 49,283'' N	rural area
RW12	Arakil	Irañeta	1º 36' 3,279'' W	42º 59' 28,331'' N	rural area
RW13	Queiles	Novallas	1º 41' 18,153'' W	41º 56' 56,288'' N	rural area
RW14	Alhama	Alfaro	1º 45' 39,234'' W	42º 10' 41,501'' N	rural area
RW15	Ega	Estella	2º 0' 43,720'' W	42º 38' 57,873'' N	upstream WWTP
RW16	Ega	Estella	2º 0' 40,040'' W	42º 38' 52,060'' N	downstream WWTP
RW17	Ega	Pamplona	1º 43' 6,810'' W	42º 48' 38,690'' N	upstream WWTP
RW18	Arga	Pamplona	1º 43' 11,970'' W	42º 48' 44,950'' N	downstream WWTP
RW19	Ebro	Tudela	1º 34' 15,510'' W	42º 3' 20,390'' N	upstream WWTP
RW20	Ebro	Tudela	1º 33' 43,410'' W	42º 3' 12,140'' N	downstream WWTP
RW21	Biadasoa	Bera de Bidasoa	1º 41' 56,640'' W	43º 16' 48,540'' N	upstream WWTP
RW22	Biadasoa	Bera de Bidasoa	1º 41' 47,420'' W	43º 16' 44,120'' N	downstream WWTP
RW23	Adour	Bayonne	1º 21' 54'' W	43º 29' 36''N	rural area
RW24	Gabas	Poursiugues-Boucoue	0º 21' 6'' W	43º 33' 16''N	rural area
RW25	Garonne	Toulouse	1º 23' 17'' E	43º 30' 41''N	rural area
RW26	Save	Le Castéra	1º 8' 60'' E	43º 39' 9''N	rural area
RW27	Echez	Maubourguet	1º 1' 53'' E	43º 28' 5''N	rural area
RW28	Baïse	Fontrailles	0º 23' 24'' E	43º 20' 35'' N	rural area

3.2. Acid pre-treatment of river water samples.

Table S2. Mean size and recovery of microparticles subjected to pre-treatments with nitric acid. Plastic microparticles: 2 μm PS (BCR). Mean ± standard deviation (n = 3).

Pre-treatment	HNO_3 (% *v/v*)	Mean size (μm)	Particle concentration ($x10^6$ L^{-1})	Particle recovery (%)
**no**	-	2.12 ± 0.02	-	-
heating (100ºC 5 min)				
	0.2	2.12 ± 0.03	189 ± 12	60
	1	2.12 ± 0.02	218 ± 13	69
	10	2.12 ± 0.02	207 ± 9	66
	50	2.04 ± 0.02	86 ± 17	27
without heating (room temperature, 24h)				
	10	2.20 ± 0.01	220 ± 5	65

Figure S1. Time scan corresponding to the analysis of bacteria *E.coli* labelled with Ag. (a) corresponds to the bacteria time scan without the treatment. (b) corresponds to the bacteria time scan with the treatment.

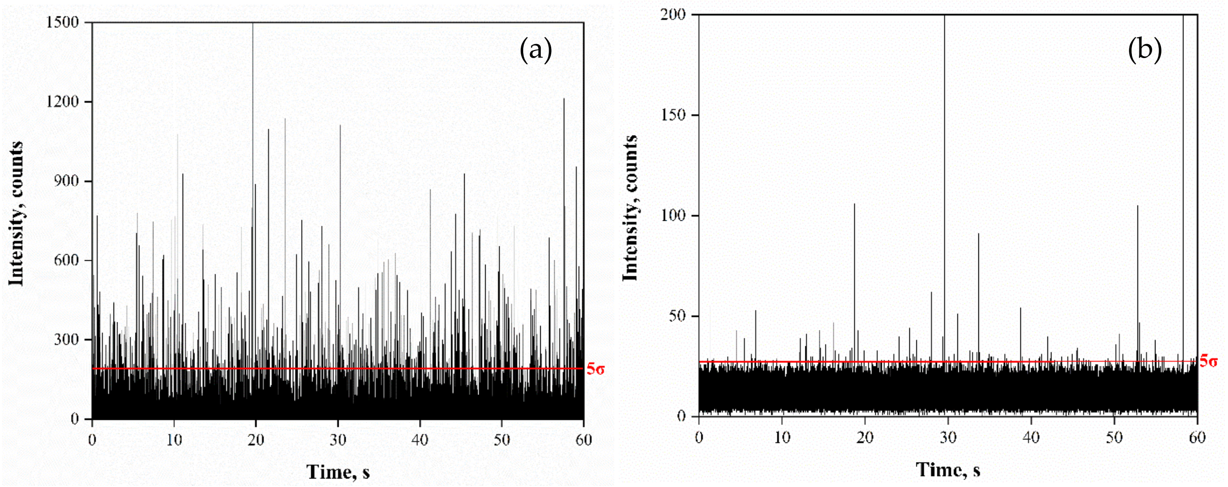

Figure S2. FESEM image of bacteria form river water filtered on glass fibre.

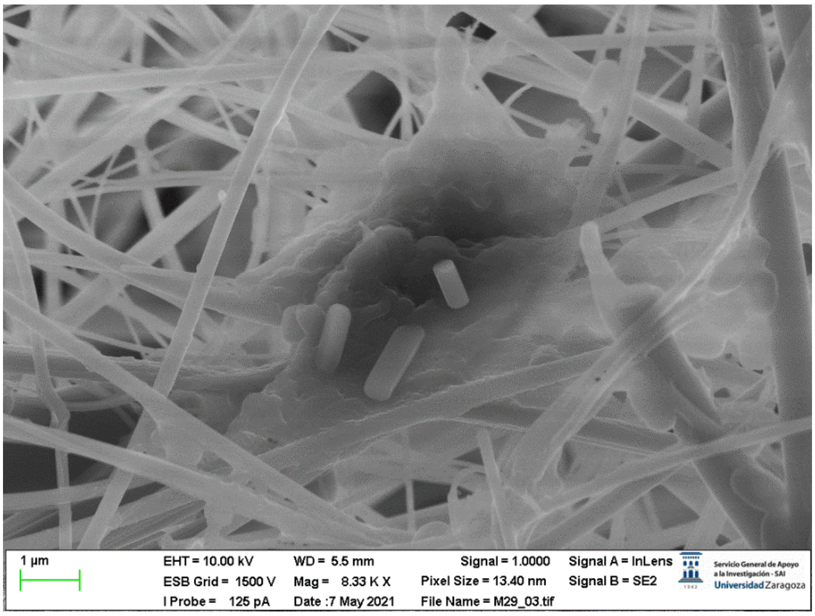

3.3. Analysis of river water samples by scanning electron and Raman microscopies.

Figure S3. Raman spectra of the plastic particles presented in Table 5. In black, spectra obtained from the analysis of the river water particles. In red spectra of the most probable compositions provided by the software KnowItAll™.

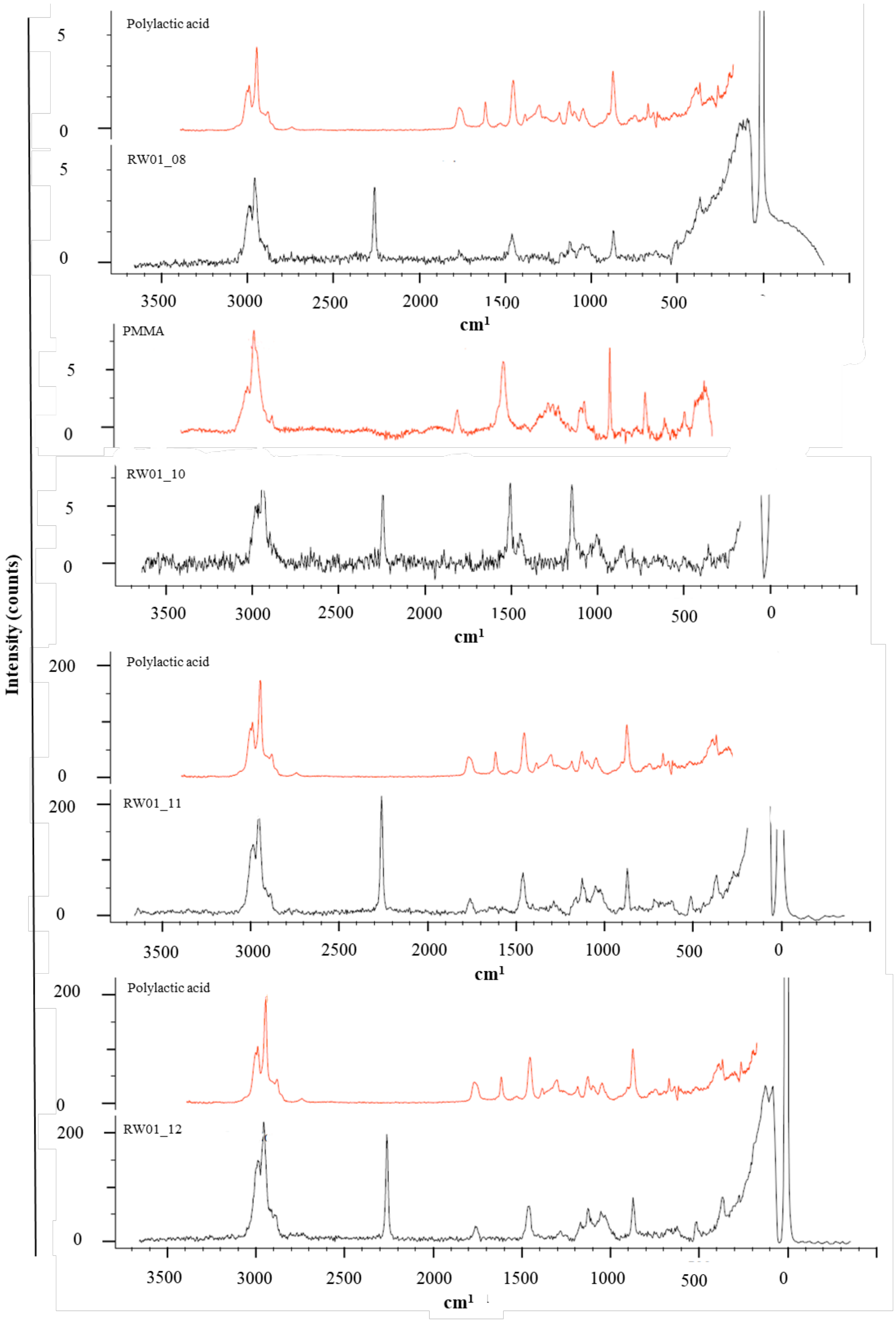

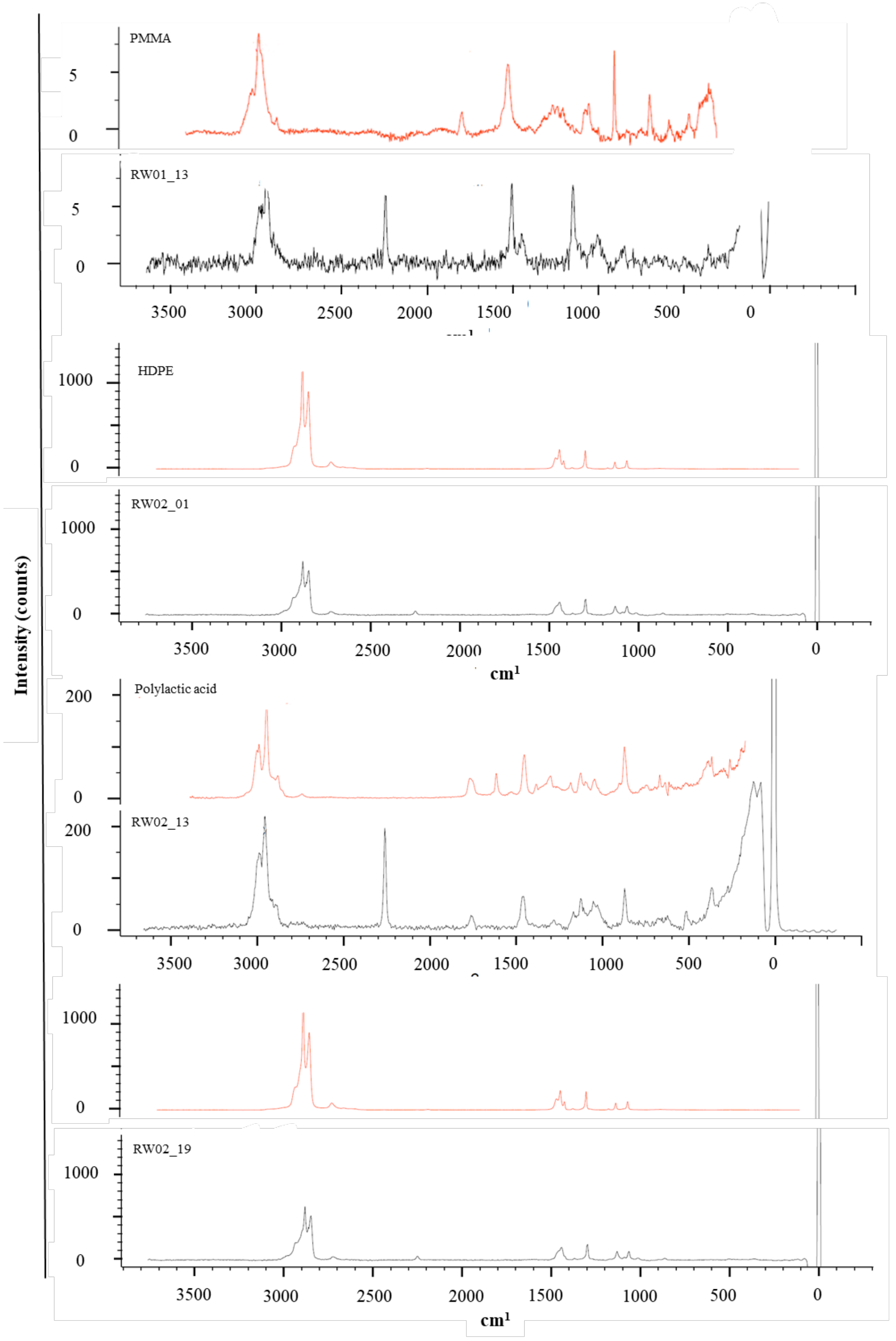

Intensity (counts)
PMMA
5
0
RW01_13
5
0
3500
3000
2500
2000
1500
1000
500
0
HDPE
1000
0
RW02_01
1000
0
3500
3000
2500
2000
1500
1000
500
0
cm1
Polylactic acid
200
0
RW02_13
200
0
3500
3000
2500
2000
1500
1000
500
0
1000
0
RW02_19
1000
0
3500
3000
2500
2000
1500
1000
500
0
cm1

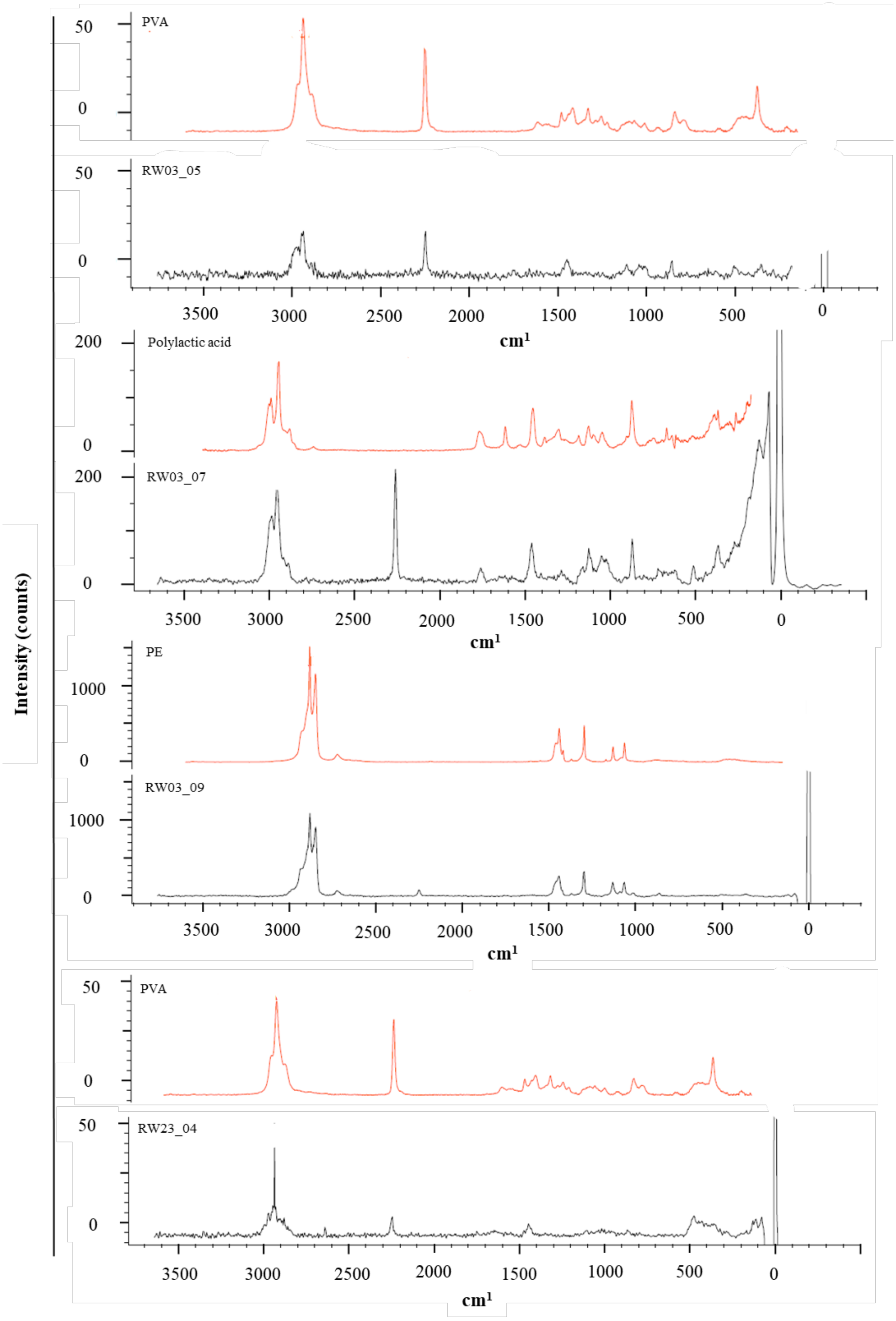
Intensity (counts)
PVA
RW03_05
Polylactic acid
RW03_07
PE
RW03_09
PVA
RW23_04
cm1
50
0
200
1000
3500
3000
2500
2000
1500
1000
500

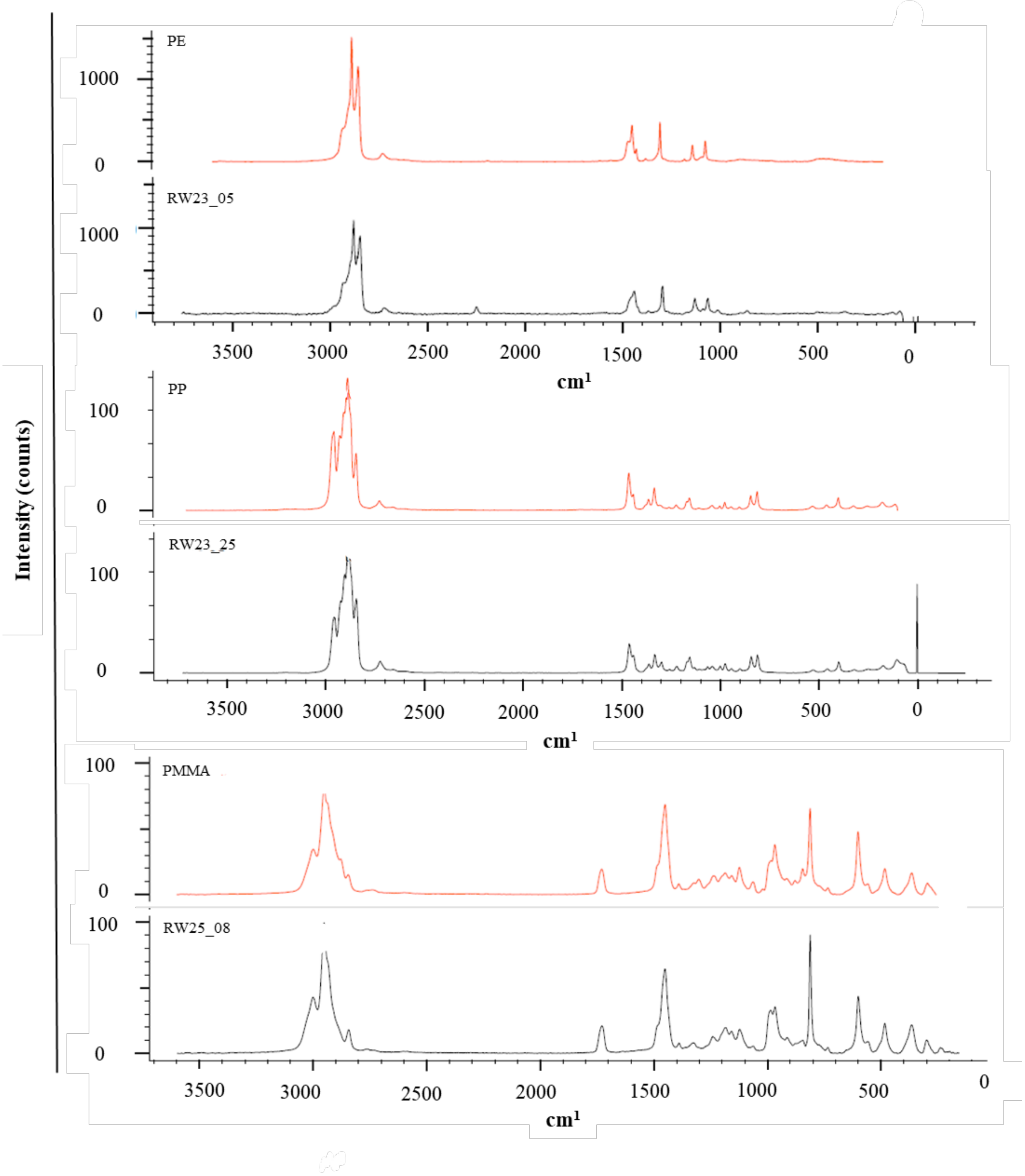

Intensity (counts)
PE
1000
0
RW23_05
1000
0
3500
3000
2500
2000
1500
1000
500
0
cm1
PP
100
0
RW23_25
100
0
3500
3000
2500
2000
1500
1000
500
0
cm1
PMMA
100
0
RW25_08
100
0
3500
3000
2500
2000
1500
1000
500
0
cm1
